# Case Report: Lupus miliaris disseminatus faciei successfully treated with thalidomide combined with photodynamic therapy: two cases and literature review

**DOI:** 10.3389/fimmu.2026.1724732

**Published:** 2026-02-25

**Authors:** Haixia Feng, Zhongbin Sun, Hao Li, Changxu Han, Zhenying Zhang

**Affiliations:** 1 Department of Dermatology, The University of Hong Kong-Shenzhen Hospital, Shenzhen, China; 2 Department of Dermatology, General Hospital of Xinjiang Military Command, Urumqi, China

**Keywords:** *Lupus miliaris disseminatus faciei* (LMDF), pathogenesis, photodynamic therapy, thalidomide, facial

## Abstract

Lupus miliaris disseminatus faciei (LMDF) is a rare granulomatous rosacea characterized by multiple papular eruptions primarily affecting the central face, resulting in both physical discomfort and notable cosmetic concerns. Despite various proposed therapies, the optimal treatment for LMDF remains controversial. Photodynamic therapy (PDT) employs a photosensitizer, light source, and oxygen to generate reactive oxygen species (ROS) that selectively target and destroy abnormal tissue, and it has also been shown to reduce and prevent scar formation. However, data on PDT use in LMDF is limited. Here, we report two young male patients with LMDF presenting with multiple firm red papules on the face, both of whom had previously received four lines of treatment—including antibiotics (minocycline, doxycycline, clarithromycin), topical tacrolimus, and traditional Chinese medicine—without clinical improvement. Both patients subsequently underwent four sessions of 5-aminolevulinic acid-based PDT (5-ALA-PDT) combined with oral thalidomide. Following this combination therapy, the lesions showed marked resolution, leaving only minimal depressed scars, and no relapse occurred during a 24-month follow-up. These cases suggest that PDT combined with thalidomide may provide an effective option for refractory LMDF, with early intervention potentially minimizing scarring. Further studies and controlled trials are warranted to confirm the efficacy and safety of this therapeutic approach.

## 1 Introduction

Lupus miliaris disseminatus faciei (LMDF) is a rare, chronic granulomatous dermatosis of uncertain etiology. First described by Fox in 1878 as “disseminated follicular lupus”, clinically, it manifests as symmetric, erythematous to yellow - brown papules (2–4 mm in diameter) predominantly on the face, often resolving with atrophic scarring (1). LMDF primarily affects young adults (20–40 years), with a slight male predominance, though cases in children and the elderly have been reported (2).

Histopathologically, early - stage lesions demonstrate tuberculoid granulomas with central caseous necrosis, while late - stage lesions exhibit perifollicular fibrosis (3). Despite its benign nature, LMDF poses significant cosmetic and psychological burdens due to its chronic course and scarring sequelae.

The pathogenesis of LMDF remains enigmatic. While early theories implicated Mycobacterium tuberculosis infection, molecular studies have consistently refuted this link (4). Current hypotheses focus on aberrant immune responses to follicular components or commensal bacteria. Notably, Propionibacterium acnes DNA has been detected within granulomas, suggesting its role in triggering a necrotizing immune reaction (5). Additionally, Demodex mites and hypersensitivity to keratin or sebum have been proposed as contributors (6). These mechanisms align with LMDF’s histopathological features, including follicular destruction and granulomatous inflammation (7).

Therapeutic management of LMDF is challenging due to the lack of standardized guidelines. First - line treatments include tetracyclines (e.g., doxycycline), which exert anti - inflammatory effects but show variable efficacy (30–50% improvement) and require prolonged administration (8). Systemic corticosteroids, though effective in early stages, are limited by adverse effects such as adrenal suppression and osteoporosis (9). Isotretinoin, a retinoid targeting sebaceous glands, achieves partial remission in some cases but carries risks of teratogenicity and xerosis (10). Dapsone, an anti - inflammatory agent, demonstrates moderate efficacy (60–80% remission) but may induce hemolytic anemia (11). Given these limitations, novel therapies are urgently needed.

Photodynamic therapy (PDT) and immunomodulators like thalidomide have emerged as promising alternatives. PDT utilizes a photosensitizer (e.g., 5 - aminolevulinic acid, 5 - ALA) activated by specific light wavelengths to generate reactive oxygen species (ROS), selectively destroying hyperproliferative cells and P. acnes while minimizing scarring (12). Thalidomide, a potent immunomodulator, inhibits TNF-α production, suppresses neutrophil chemotaxis, and shifts Th1/Th2 balance, making it effective in granulomatous disorders (13). Combining these modalities may synergistically target both inflammatory and microbial components of LMDF, offering a paradigm shift in refractory cases. Herein, we present two cases successfully treated with PDT in combination with thalidomide.

## 2 Case description

This study was granted an ethical exemption by the Ethics Committee of The University of Hong Kong–Shenzhen Hospital (HKUSZH2025098). All procedures were conducted in accordance with ethical standards to ensure the protection of participants’ rights and confidentiality. Written informed consent was obtained from both participants.

### 2.1 Patient 1

**Figure 1:**
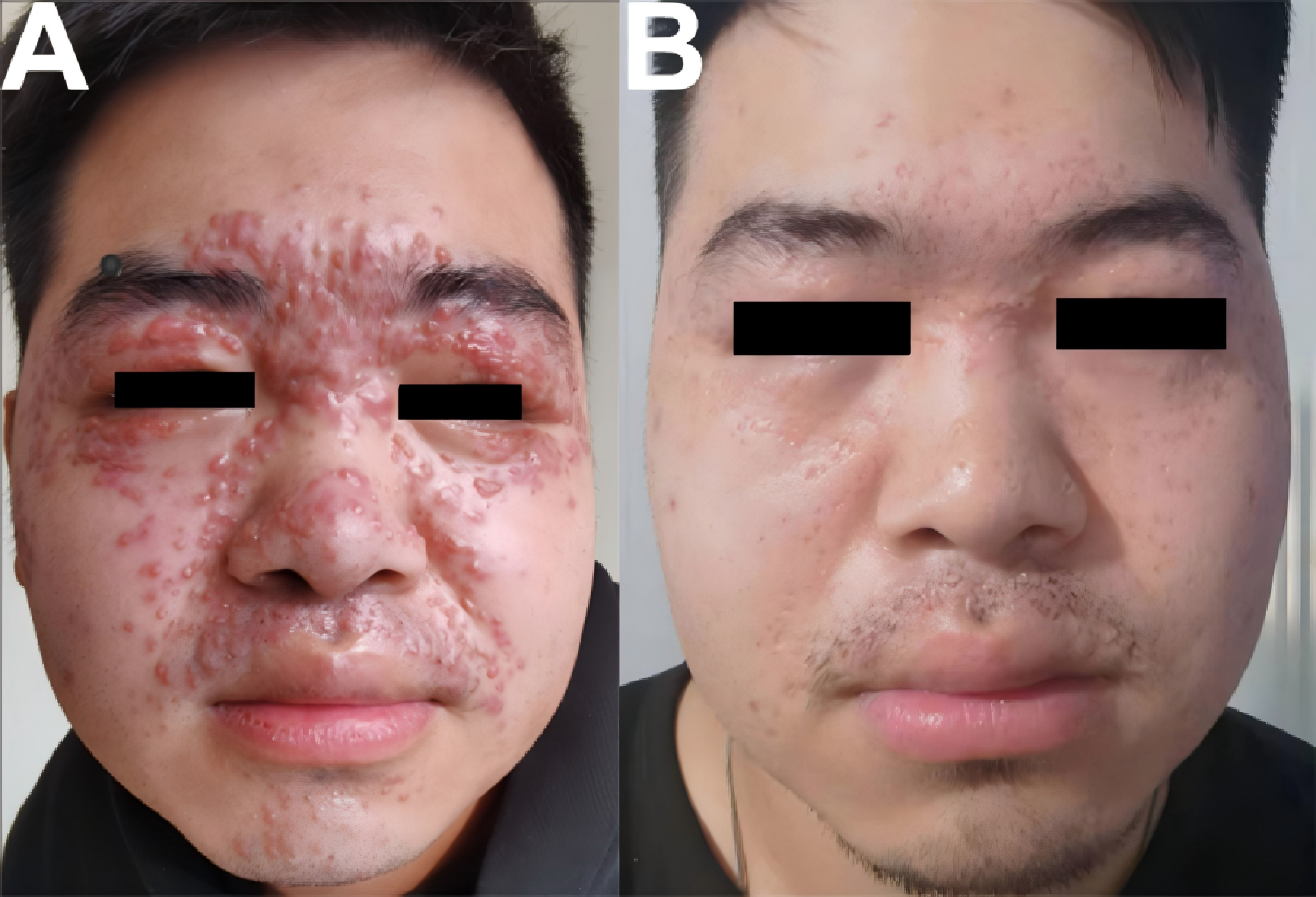
**(A)** On dermatological examination, multiple and densely distributed round papules, measuring 3–5 mm in size, were observed on the face. These papules ranged in color from red to dark red, with distinct boundaries. In some of the rashes, a horny plug was visible at the center. The rashes were most prominent around the eyes, nose, and mouth. **(B)** Follow-up Images (Six Months after the Completion of Thalidomide Combined with Photodynamic Therapy).

**Figure 2:**
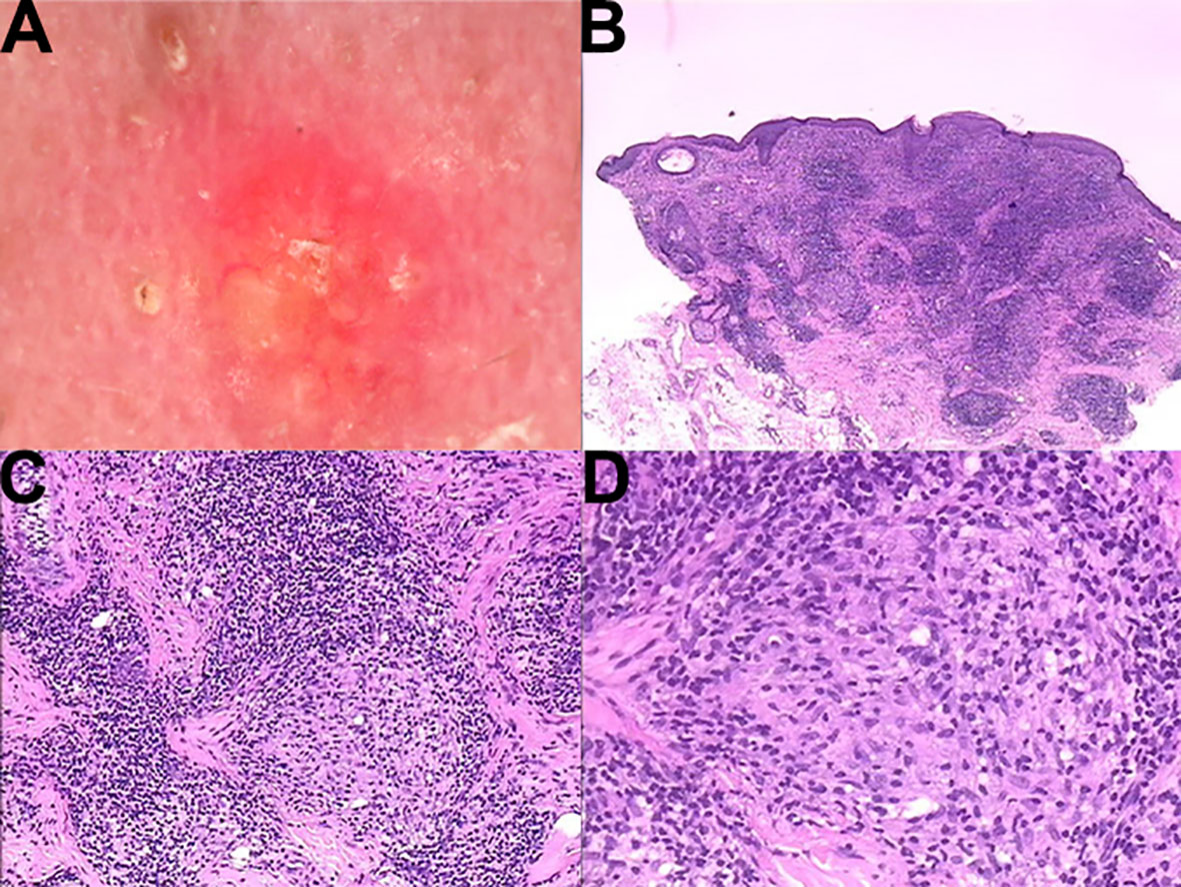
**(A)** Dermoscopy revealed target - like follicular horn plugs on a red - yellow translucent homogeneous structure. **(B)** 10×, **(C)** 20×, **(D)** 40× Histopathological examination demonstrated that diffuse mixed inflammatory cell infiltration was visible throughout the entire dermis. In the dermis, there were numerous massive epithelioid granuloma masses. Lymphocytes surrounded these masses, and histiocytes could be observed in the center.

A 23-year-old male patient presented with a chief complaint of multiple red papules on the face persisting for more than 11 months. Since disease onset, he reported no pruritus, pain, or other subjective discomfort. There was no history of trauma, and his past medical history was unremarkable, with no family history of similar conditions. As shown in **Figure 1A**, clinical examination revealed multiple well-demarcated red to dark-red papules measuring 3–5 mm in diameter on the face, predominantly distributed around the periorbital, nasal, and perioral regions. Central keratin plugs were observed in some lesions. Dermoscopic examination demonstrated target-like follicular horn plugs on a red–yellow translucent homogeneous background. Histopathological analysis revealed numerous epithelioid granulomas in the superficial and mid-dermis, accompanied by increased perigranulomatous lymphocytic infiltration (**Figure 2**). Based on these findings, a final diagnosis of lupus miliaris disseminatus faciei (LMDF) was established.

Prior to presentation at our hospital, the patient had been treated at an outside institution with tanshinone capsules (four capsules, three times daily for 2 weeks) combined with topical isotretinoin–erythromycin gel (twice daily), without clinical improvement. Subsequently, he received Jiedu Sanjie capsules (two capsules, three times daily for 3 weeks) in combination with topical fusidic acid cream (twice daily), which also proved ineffective.

After referral to our hospital, treatment was initiated with clarithromycin sustained-release tablets (0.5 g once daily for 4 weeks), combined with 0.03% tacrolimus ointment (twice daily) and red–blue light therapy administered every 3 days. However, the skin lesions failed to improve and showed mild progression. The therapeutic regimen was then switched to doxycycline tablets (0.1 g twice daily for 6 weeks), combined with triamcinolone acetonide–econazole cream (once daily) and fusidic acid cream (once daily). During this period, the lesions remained stable, with neither improvement nor deterioration.

The patient was subsequently treated with thalidomide tablets (75 mg nightly for 1 week, followed by 100 mg nightly for 3 weeks; total duration: 4 weeks). After this course, the lesions appeared slightly darker in color, with a modest reduction in size. Given the partial response, the treatment strategy was modified to include photodynamic therapy (PDT) (four sessions, once every 2 weeks) combined with thalidomide (100 mg nightly for 4 weeks, followed by 50 mg nightly for an additional 2 weeks). Contraception was advised during thalidomide treatment and for 4 weeks after discontinuation (**Table 1**).

**Table 1:** Treatment progress and follow-up data.

Parameter	Patient 1	Patient 2
Age/Sex	23/M	28/M
Duration of Lesions	11 months	2 years
Previous Treatments	1. Tanshinone capsules, isotretinoin erythromycin gel 2. Jiedu Sanjie capsules, fusidic acid cream 3. Clarithromycin, tacrolimus, red-blue light therapy 4. Doxycycline, triamcinolone acetonide econazole cream, fusidic acid cream	1. Xiaocuo pills, mupirocin ointment 2. Compound glycyrrhizin tablets, mometasone furoate cream 3. Doxycycline, adapalene gel, fusidic acid cream 4. Clarithromycin, methylprednisolone, tacrolimus, fusidic acid cream
Thalidomide Dose	75–100 mg qd (tapered to 50 mg)	100 mg qd (tapered to 50 mg)
PDT Sessions	4 sessions (biweekly)	4 sessions (biweekly)
Lesion Reduction	95% after 8 weeks	90% after 8 weeks
Adverse Effects	Mild erythema, tolerable pain	Mild erythema, transient folliculitis
Follow-up (3 months)	Residual atrophic scars	Residual atrophic scars

Prior to PDT, the patient provided written informed consent, underwent facial cleansing, and standardized clinical photographs were obtained. A 5-aminolevulinic acid (5-ALA) solution at a concentration of 7.5–10% (prepared from 118 mg 5-ALA; Shanghai Fudan–Zhangjiang Bio-Pharmaceutical Co., Ltd.) was applied. To enhance drug penetration, intact lesions were vertically punctured using a 2.5 mL syringe needle, with gentle removal of jelly-like contents. The prepared 5-ALA solution was then evenly applied to the lesions, which were subsequently occluded and protected from light for 3 hours. After incubation, the patient’s face was cleansed again.

Photodynamic therapy was performed using a high-energy LED red-light device (wavelength: 633 nm; Shanghai Fudan–Zhangjiang Bio-Pharmaceutical Co., Ltd.), with an energy density of 60–70 J/cm². The light source was positioned approximately 10 cm from the skin surface, and irradiation was delivered for a total duration of 20 minutes. Detailed PDT parameters are summarized in **Table 2**.

**Table 2:** The parameters of photodynamic therapy protocol.

Session	5-ALA concentration (%)	occlusion time (h)	light dose (J/cm²)	light exposure period (min)
Patient 1#				
session 1	7.5	3	60	20
session 2	10	3	60	20
session 3	10	3	60	20
session 4	10	3	70	20
Patient 2#				
session 1	7.5	3	60	20
session 2	10	3	60	20
session 3	10	3	60	20
session 4	10	3	70	20

Pain intensity during each PDT session was systematically evaluated using the Numeric Rating Scale (NRS). During the initial 5 minutes of irradiation at 60 J/cm², the patient reported minimal discomfort (NRS score: 0). During the subsequent 15 minutes, when the energy density was increased to 70 J/cm², the NRS score increased slightly to 1. Overall, the discomfort was mild and well tolerated, and no treatment interruption or discontinuation occurred during any of the four PDT sessions.

To further alleviate heat sensation and light-induced pain, continuous air cooling was applied throughout the entire PDT procedure using a standard tabletop electric fan positioned approximately 30–50 cm from the treatment area. The airflow was adjusted to a low-to-moderate speed according to patient comfort, providing a noticeable cooling effect without causing additional discomfort. No topical or systemic anesthetic or analgesic agents were administered before or during PDT.

After completion of four PDT sessions combined with thalidomide therapy, approximately 95% of the lesions resolved, leaving scattered 2–3 mm residual papules and multiple 3–4 mm depressed scars (**Figure 1B**). Transient erythema and swelling were observed after PDT, which were managed with a hyaluronic acid patch (Winona, Kunming, China) and topical human epidermal growth factor (hEGF; GeneTime, Shenzhen, China).

No leukopenia, peripheral neuropathy, or other systemic adverse effects were observed during treatment. Six months after completion of therapy, the patient underwent fractional CO_2_ laser treatment for residual depressed scars. Follow-up evaluations at 6, 12, and 24 months after treatment completion revealed no evidence of disease relapse.

### 2.2 Patient 2

A 28-year-old male patient presented with a 2-year history of multiple red papules on the face. Since disease onset, he reported no pruritus, pain, or other subjective discomfort, and there was no history of trauma. His past medical history was unremarkable, and no family history of similar conditions was reported. Notably, the patient resided in the same village as Patient 1; however, no additional cases had been identified in that area.

**Figure 3:**
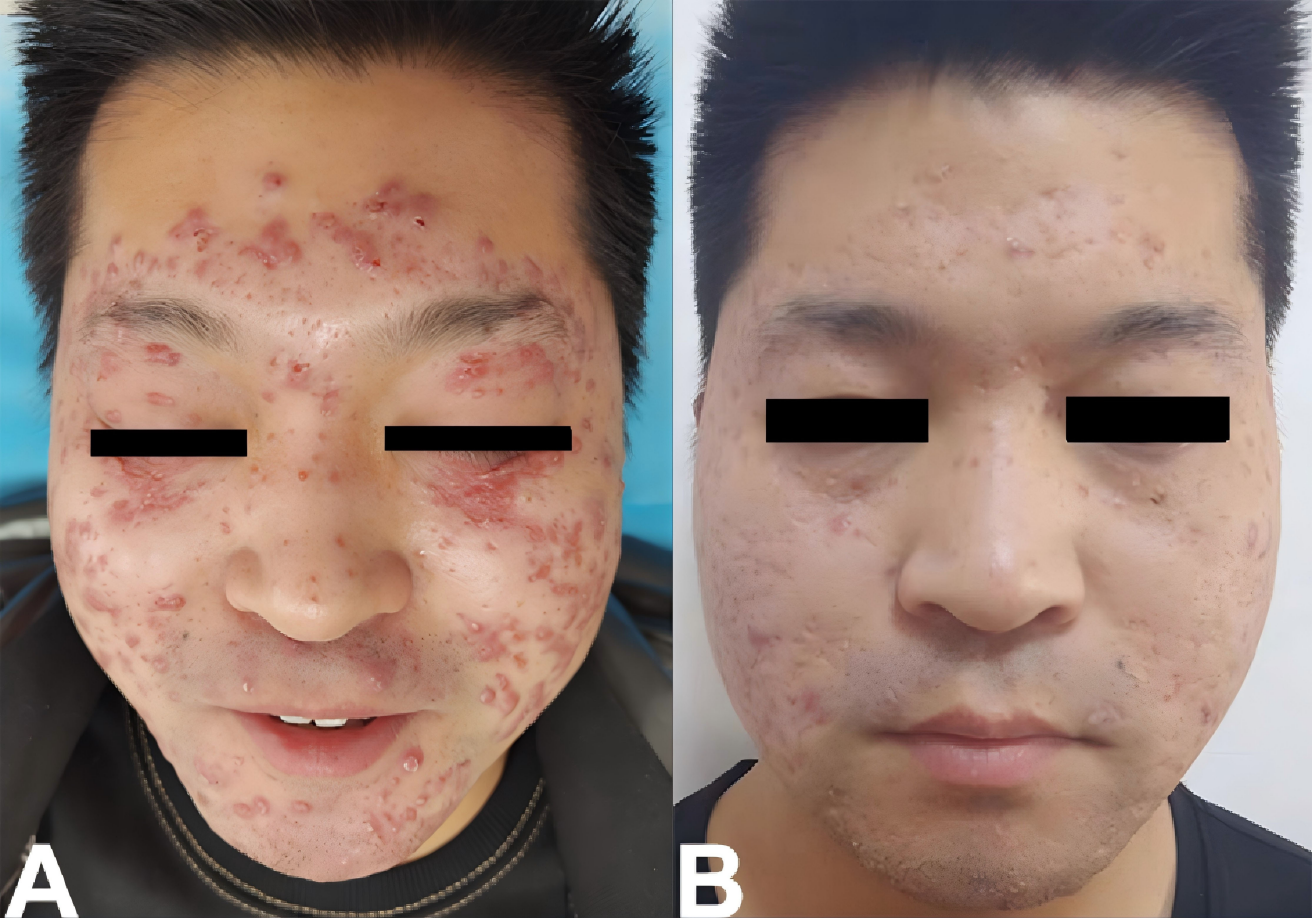
**(A)** The clinical manifestations presented as multiple round red papules, measuring 3–5 mm in size, on the face. These papules had distinct boundaries, with a horny plug visible at the center. The rashes were particularly prominent on the forehead, around the eyes, around the mouth, and on the cheeks. Moreover, numerous depressed scars and cord-like scars were observable. **(B)** Follow-up Images (Six Months after the Completion of Thalidomide Combined with Photodynamic Therapy).

**Figure 4:**
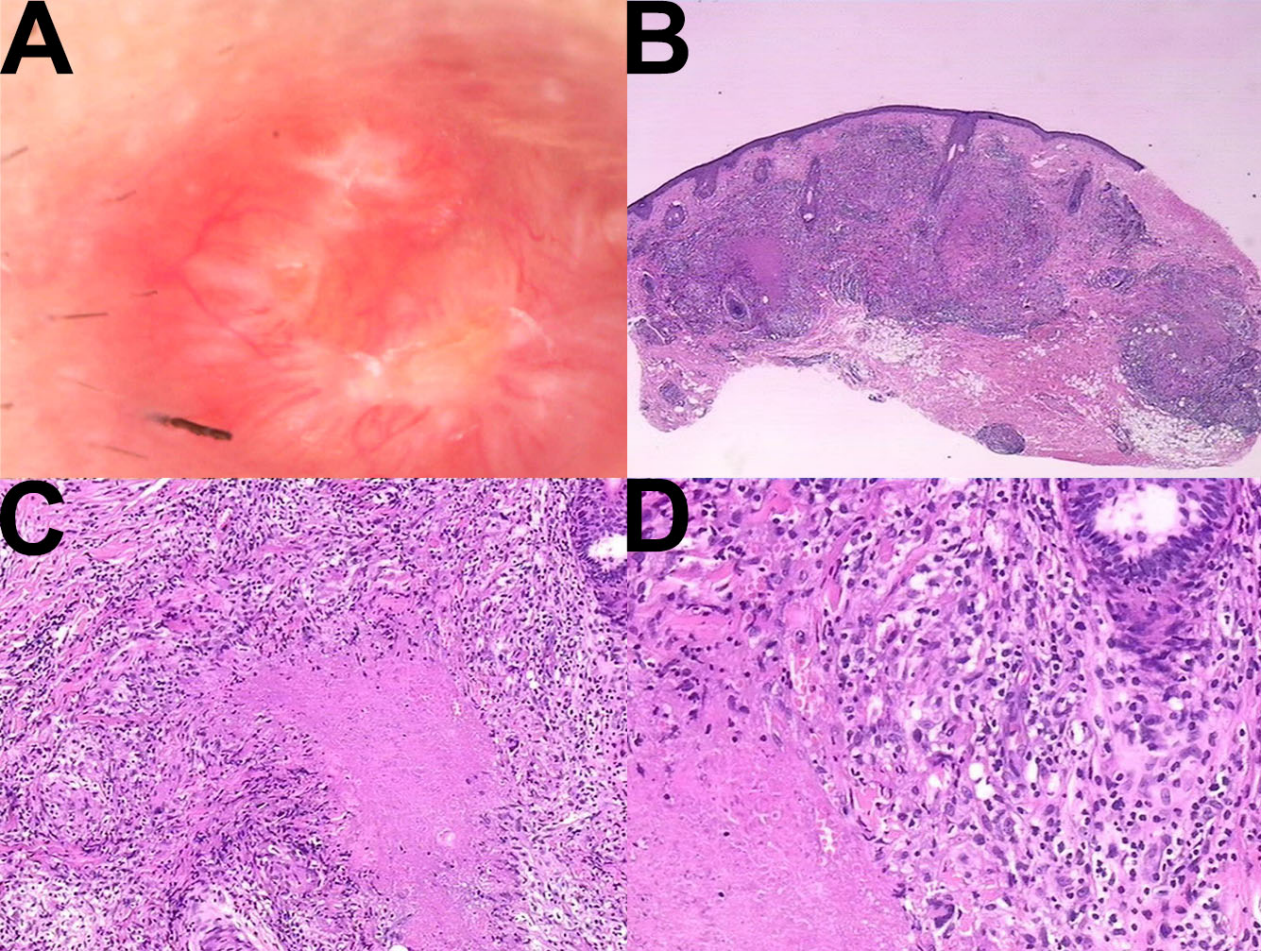
**(A)** Dermoscopy revealed target - like follicular horn plugs on a red - yellow translucent homogeneous structure, and scar formation was visible. **(B)** 10×, **(C)** 20×, **(D)** 40× Histopathological examination showed a mixed inflammatory cell infiltration throughout the entire layer of the dermis. In some areas, tuberculoid granulomas were observed. The center of the nodules consisted of amorphous caseous substances. Histiocytes were visible surrounding the caseous substances, and the periphery was infiltrated by a relatively large number of lymphocytes and neutrophils.

As shown in **Figure 3A**, clinical examination revealed multiple well-demarcated red papules measuring 3–5 mm in diameter on the face, accompanied by scattered depressed scars. Dermoscopic evaluation demonstrated target-like follicular horn plugs on a red–yellow translucent homogeneous background, with evidence of scar formation. Histopathological examination revealed epithelioid granulomas with central caseous necrosis in the superficial and mid-dermis, along with increased perigranulomatous lymphocytic infiltration (**Figure 4**). Based on these clinical, dermoscopic, and histopathological findings, a definitive diagnosis of lupus miliaris disseminatus faciei (LMDF) was established.

Comprehensive laboratory and auxiliary investigations, including routine blood and urine tests, liver and renal function tests, antinuclear antibody profile, erythrocyte sedimentation rate, purified protein derivative (PPD) test, chest radiography, and electrocardiography, yielded normal results.

Prior to presentation at our hospital, the patient had received multiple unsuccessful treatments at an outside institution. Initial therapy consisted of oral traditional Chinese medicine decoctions (details unavailable) for 3–4 months, without clinical improvement. Treatment was subsequently switched to Xiaocuo pills (one packet, three times daily for 4–6 weeks) combined with topical mupirocin ointment (twice daily), which was also ineffective.

The patient was then prescribed compound glycyrrhizin tablets (two tablets, three times daily for 2 weeks) in combination with 0.1% mometasone furoate cream, without noticeable improvement. The regimen was further modified to doxycycline tablets (0.1 g twice daily for 2 weeks), along with topical adapalene gel (once nightly) and fusidic acid cream (twice daily); however, the lesions remained unresponsive.

Subsequently, treatment was adjusted to clarithromycin sustained-release tablets (0.5 g once daily for 6–8 weeks), combined with oral methylprednisolone (12 mg once daily for 2 weeks), together with topical fusidic acid ointment (once daily) and tacrolimus ointment (once daily). Despite this comprehensive regimen, no significant clinical improvement was observed.

Upon referral to our hospital, treatment was initiated with thalidomide tablets (100 mg nightly for 4 weeks, followed by 50 mg nightly for an additional 2 weeks; total duration: 6 weeks) in combination with four sessions of photodynamic therapy (PDT), administered once every 2 weeks (**Table 1**). Written informed consent was obtained prior to treatment. The patient underwent facial cleansing and standardized clinical photography before PDT. A 5-aminolevulinic acid (5-ALA) solution (118 mg; Shanghai Fudan–Zhangjiang Bio-Pharmaceutical Co., Ltd.) was prepared at a concentration of 7.5–10% and applied to the lesions.

To enhance drug penetration, intact lesions were vertically punctured using a 2.5 mL syringe needle, with gentle removal of jelly-like contents. The prepared 5-ALA solution was evenly applied to the lesions, which were then occluded and protected from light for 3 hours. After incubation, the patient cleansed the face, followed by irradiation with a high-energy LED red-light device (wavelength: 633 nm; Shanghai Fudan–Zhangjiang Bio-Pharmaceutical Co., Ltd.). The energy density was set at 60–70 J/cm², with the light source positioned approximately 10 cm from the skin surface, and irradiation delivered for 20 minutes. Detailed PDT parameters are summarized in **Table 2**.

The PDT protocol, including pain assessment and management, was identical to that used for Patient 1. Pain intensity during each session was assessed using the Numeric Rating Scale (NRS). During the initial 5 minutes of irradiation at 60 J/cm², the patient reported minimal discomfort (NRS score: 1). During the subsequent 15 minutes, when the energy density was increased to 70 J/cm², the pain intensity increased slightly to an NRS score of 2. Overall, the patient tolerated all treatment sessions well, and no interruption or discontinuation was required.

To mitigate light-induced heat sensation and pain, continuous air cooling was applied throughout the irradiation period using a standard tabletop electric fan positioned approximately 30–50 cm from the treatment area. The airflow was adjusted to a low-to-moderate speed based on patient comfort. No topical or systemic anesthetic or analgesic agents were administered before or during PDT.

After completion of four PDT sessions combined with six weeks of thalidomide therapy, approximately 90% of the lesions resolved, leaving scattered 3–4 mm residual papules, multiple erythematous areas, and depressed scars (**Figure 3B**). Contraception was advised during thalidomide treatment and for 4 weeks after drug discontinuation. Throughout the treatment period, no leukopenia, peripheral neuropathy, or other systemic adverse events were observed. Follow-up evaluations at 6, 12, and 24 months after treatment completion demonstrated no evidence of disease recurrence.

## 3 Discussion

The etiology and pathogenesis of lupus miliaris disseminatus faciei (LMDF) remain incompletely understood despite extensive investigation. Early hypotheses implicating *Mycobacterium tuberculosis* infection have not been supported by molecular evidence (7), prompting a shift toward alternative concepts, including aberrant immune responses involving the folliculosebaceous unit and a possible relationship with the rosacea spectrum (14). The detection of *Propionibacterium acnes* within LMDF granulomas further suggests a role for microbial antigens in triggering necrotizing granulomatous inflammation, although its precise pathogenic significance remains uncertain (5).

The diagnosis of LMDF is often challenging, particularly when patients are reluctant to undergo facial biopsy due to concerns about scarring (15). In this context, dermoscopy provides a valuable non-invasive adjunct, enabling recognition of characteristic features such as target-like follicular keratin plugs and linear or hairpin-shaped vessels (16, 17). In our cases, dermoscopic findings were highly consistent with histopathological features, supporting its utility in guiding diagnosis and management in clinical practice.

Therapeutic management of LMDF remains difficult because of the lack of standardized treatment guidelines and the variable efficacy of existing therapies. Conventional oral medications, including tetracycline antibiotics, dapsone, isotretinoin, corticosteroids, and quinacrine, have shown variable efficacy (18). The requirement for long - term treatment, often exceeding 12 weeks, not only tests the patient’s compliance but also increases the risk of adverse effects. Tetracycline antibiotics, for example, have been used for years with both successful and failed outcomes, along with reports of adverse drug reactions (9). Dapsone, while cost - effective and safe, has an unclear mechanism of action (12). Isotretinoin, despite its anti - inflammatory and sebaceous - gland - modulating properties, has potential side effects that necessitate careful monitoring (11).

The remarkable efficacy observed in our cases (90–95% lesion clearance) likely stems from the complementary mechanisms of thalidomide and PDT. Thalidomide exerts potent immunomodulatory effects through inhibition of TNF-α production, suppression of neutrophil recruitment, and attenuation of granuloma formation, thereby addressing the immune dysregulation underlying LMDF (19). Concurrently, PDT targets P. acnes via 5 - ALA - induced protoporphyrin IX (PpIX), which generates cytotoxic ROS upon red light irradiation (630 nm). This dual approach not only eradicates bacterial triggers but also disrupts hyperactive follicular units, preventing lesion recurrence. Notably, microneedling - enhanced 5 - ALA penetration ensured precise targeting of sebaceous glands, amplifying therapeutic effects (20).

Our results surpass those of conventional therapies (**Table 3**). Tetracyclines, while reducing inflammation, achieve only partial remission (50% improvement) and require months of treatment (8). Systemic corticosteroids, though rapid - acting, are unsuitable for chronic use due to systemic toxicity (9). In contrast, the thalidomide - PDT combination achieved near - complete resolution within 10 weeks, with minimal adverse effects (transient erythema). This aligns with prior studies reporting PDT’s scar - sparing benefits in acne vulgaris and granulomatous rosacea (21), and with the established efficacy of thalidomide in discoid lupus erythematosus (16).

**Table 3:** Comparative analysis of previous studies vs. current cases.

Study	Treatment	Sample size	Efficacy	Adverse effects
Al-Mutairi (2011) (7)	Tetracyclines	12 patients	50% improvement	Gastrointestinal upset
Kumano et al. (1983) (11)	Dapsone + prednisone	5 patients	80% remission	Hemolytic anemia
Bahadir et al. (1998) (10)	Isotretinoin	2 patients	Partial response	Xerosis, teratogenicity
Current Cases	Thalidomide + PDT	2 patients	90–95% remission	Mild erythema, no severe AE

This study has several limitations, including the small sample size and the uncontrolled design, which preclude definitive conclusions regarding efficacy and long-term recurrence risk. Nevertheless, the sustained clinical remission observed during extended follow-up and the absence of serious adverse events support the potential feasibility of this combination approach. Further studies involving larger cohorts and mechanistic analyses are warranted to confirm these preliminary observations and to better define the role of thalidomide combined with ALA-PDT in the management of refractory LMDF.

In conclusion, this report describes the first documented use of thalidomide combined with 5-aminolevulinic acid–based photodynamic therapy in the treatment of refractory lupus miliaris disseminatus faciei. The combination therapy resulted in rapid and sustained clinical improvement with favorable tolerability and no disease relapse during long-term follow-up. By simultaneously targeting immune dysregulation and follicular inflammatory components, this approach may represent a promising therapeutic option for patients with treatment-resistant LMDF. Although further validation in larger, controlled studies is required, our findings provide preliminary clinical evidence supporting the potential role of thalidomide–PDT combination therapy in the management of this challenging condition.

## Data Availability

The raw data supporting the conclusions of this article will be made available by the authors, without undue reservation.
